# Characteristics of Polysaccharides from Industrial Hemp (*Cannabis sativa* L.) Kernels

**DOI:** 10.3390/foods13213429

**Published:** 2024-10-28

**Authors:** Ping Wei, Yayuan Tang, Kui Zhou, Zhen Wei, Guoming Liu

**Affiliations:** 1 Agro-Food Science and Technology Research Institute, Guangxi Academy of Agricultural Sciences, 174 East Daxue Road, Nanning 530007, China; weiping@gxaas.net (P.W.); tangyayuan@gxaas.net (Y.T.); zhoukui@gxaas.net (K.Z.); zhenwei@gxaas.net (Z.W.); 2 Guangxi Key Laboratory of Fruits and Vegetables Storage-Processing Technology, 174 East Daxue Road, Nanning 530007, China; 3 Guangxi Academy of Agricultural Sciences, 174 East Daxue Road, Nanning 530007, China

**Keywords:** industrial hemp kernel, polysaccharide, antioxidant activity, structural identification

## Abstract

Polysaccharides from hemp seeds exhibit antioxidant activities in vitro and in vivo. However, crude polysaccharide quality is often low owing to the presence of fibres and pigment impurities, which are difficult to eliminate in the hemp seed shell. In this study, crude polysaccharides from hemp kernels (HKP) were obtained by hot water extraction and separated by membrane ultrafiltration into eight fractions with different molecular weights. Total antioxidant capacity and free radical scavenging (DPPH) assays were performed to evaluate the antioxidant activities of HKP and the fractions in vitro. The structural characteristics of HKP were determined using various analytical techniques. The Fe^3+^-reducing power of HKP was 7.65 ± 0.22 μmol/g, and HKP possessed the highest DPPH radical-scavenging rates (94.30 ± 2.27%), similar to 5 mg/mL Vitamin C (Vc), which had a rate of 95%. The HKP was an acidic polysaccharide with a low molecular weight (4.21 ± 0.12 kDa). The monosaccharide composition indicated that HKP primarily comprised mannose, ribose, glucuronic acid, galacturonic acid, glucose, galactose, arabinose, and fucose in a molar ratio of 0.96:1.95:8.27:0.98:9.46:1.69:6.10:2.82. The molar mass of HKP was distributed widely in a triple helical conformation. This study provides a scientific basis for further research on the use of hemp polysaccharides.

## 1. Introduction

Industrial hemp (*Cannabis sativa* L.) is an annual herbaceous plant that has been used by humans for thousands of years. In recent years, based on the emergence of new varieties (tetrahydrocannabinol content <0.3%) and legalisation of planting, it has received widespread attention and cultivation again due to its high economic benefits, strong ability to absorb carbon dioxide, soil improvement capability, and other characteristics. For example, the planting area of industrial hemp in the European Union increased by 60%, and the yield increased by 84.3% from 2015 to 2022 [1]. Currently, it is mainly grown in China, Central Asia, the Philippines, and Europe. The World Food and Agriculture Organization research data show that the industrial hemp cultivation area of China accounts for nearly 50% of the global cultivation area [2].

Industrial hemp has long been the focus of attention and utilisation [3,4,5,6]. Its leaves, seeds, and roots are processed into various products, such as clothing, food, medicine, animal feed, and daily chemical products [7]. Seeds are essential and are commonly used to produce proteins and oils due to their high content of these nutrients. However, some low-content substances in seeds, such as polysaccharides, have received less attention. Although the polysaccharide content in hemp seeds is usually only 10–15% [8], the value of polysaccharides in terms of health and developmental potential should not be ignored or wasted.

To date, some studies have revealed that hemp seeds have potential preventive effects against many diseases, such as constipation, hypertension, and oxidative stress [9,10,11]. Polysaccharides may play crucial roles as functional substances. Chang et al. studied the optimal extraction process for polysaccharides from industrial hemp residues and preliminarily identified that they comprised ten monosaccharides and some non-sugar components. They found that industrial hemp residual polysaccharides promoted anti-aging-related gene expression [8]. The physical and chemical properties of hemp seed polysaccharides have also received attention, and their rheological and emulsifying properties have been investigated. This is due to the potential applications of emulsifiers in the food industry. The antioxidant capacity is an important research topic. Cell and animal experiments have confirmed that hemp polysaccharides can resist H_2_O_2_-induced oxidative stress [12,13]. However, the limited research mentioned above is insufficient to promote the application of hemp seed polysaccharides in industries such as food and cosmetics. In addition, hemp seeds contain large amounts of fibres and pigments that are difficult to remove. This also affects the application of hemp seed polysaccharides in the food and cosmetic industries [14]. Therefore, further studies of hemp polysaccharides are required. This helps solve the problem of reusing by-products from hemp processing and meets people’s demand for healthy food.

In this study, hemp kernel polysaccharides (HKPs) were extracted from shelled hemp seeds after removing many fibres and pigment impurities. Polysaccharides with the highest antioxidant activity were screened by analysing the total antioxidant capacity (T-AOC) and 2,2-Diphenyl-1-picrylhydrazyl (DPPH) radical-scavenging rates. Moreover, the structural characteristics of HKP were also identified using high-performance liquid chromatography (HPLC), high performance gel permeation chromatography (HPGPC), Fourier transform infrared spectroscopy (FT-IR), and Congo red analysis. The potential applications in the food and cosmetic industries can be explored based on these data.

## 2. Materials and Methods

### 2.1. Reagents and Chemicals

Standards of mannose, ribose, rhamnose, glucuronic acid, galacturonic acid, *N*-acetyl-glucosamine, glucose, *N*-acetyl-galactose, galactose, xylose, arabinose, fucose, Folin–Ciocalteu reagent, DPPH, and *p*-nitrophenyl-β-galactopyranoside (PNPG) were purchased from Sigma-Aldrich Co. (St. Louis, MO, USA). Congo red and T-AOC assay kits were purchased from Beijing Solarbio Science and Technology Co. (Beijing, China). The protein quantification (TP) assay kit was purchased from the Nanjing Jiancheng Bioengineering Institute (Nanjing, China). Trifluoroacetic acid (TFA), methanol, sodium hydroxide (NaOH), 1-phenyl-3-methyl-5-pyrazolinone (PMP), potassium dihydrogen phosphate (KDP), and acetonitrile (ACN) were purchased from Fisher (Fair Lawn, NJ, USA). Pepsin, absolute ethanol, sodium nitrate (NaNO_3_), potassium bromide (KBr), deuterated H_2_O (D_2_O), tetramethylsilane (TMS), etc., chemicals and reagents were purchased from China National Pharmaceutical Group Co., Ltd. (Beijing, China).

### 2.2. Preparation of HKP

HKP was prepared using a modified method described by Wen et al. [12]. Briefly, 500 g of hemp kernels were crushed and extracted with 80% ethanol at a ratio of 1:2 (g/mL). They were then shaken vigorously for 20 min and centrifuged at 3000 rpm for 20 min to defat at room temperature. This process was repeated thrice. The dried sample was extracted with 750 mL of distilled water at 60 °C for 2 h and centrifuged at 4000 rpm for 30 min to collect the supernatant. The precipitate was extracted with 750 mL of distilled water three times and discarded. The three supernatants were then concentrated to 50 mL. Then, the protein was removed using 0.3% pepsin enzymatic hydrolysis at 37 °C for 3 h. The enzyme was inactivated at 95 °C for 10 min, and the solution was dialysed for 48 h and lyophilised to obtain HKP. Additionally, some HKP were separated by membrane ultrafiltration into eight fractions (HKPs) of different molecular weights (HKP-1 MW > 1000 kDa; HKP-2 1000 > MW > 500 kDa; HKP-3 500 > MW > 300 kDa; HKP-4 300 > MW > 100 kDa; HKP-5 100 > MW > 50 kDa; HKP-6 50 > MW > 30 kDa; HKP-7 30 > MW > 10 kDa; and HKP-8 10 > MW > 3 kDa) and lyophilised.

### 2.3. Chemical Composition Test

The sugar content of HKP was determined using the phenol-sulfuric acid method, according to Tang et al. [15]. The extraction yield (%) was calculated by Equation (1):

Yield (%) = weight of the dried crude polysaccharide (g)/weight of hemp kernel powder (g) × 100(1)

Additionally, the residual protein content of HKP was determined using a protein quantification (TP) assay kit.

### 2.4. Assay for Antioxidant Activity

Total antioxidant capacity (T-AOC) and DPPH scavenging activities were utilised to analyse the antioxidant properties of the HKP and its eight fractions. The T-AOC value was determined using a T-AOC assay kit based on the ability of polysaccharides to convert ferric (Fe^3+^) to ferrous (Fe^2+^) ions. DPPH radical scavenging activity was determined according to the method described by Liu et al. [16]. The only difference was that the concentration of HKP and the eight fraction samples used in this study was changed to 5 mg/mL.

### 2.5. Monosaccharide Composition

Monosaccharide composition was determined using HPLC according to the method described by Liu et al. [17]. Mannose, ribose, rhamnose, glucuronic acid, galacturonic acid, glucose, galactose, xylose, arabinose, fucose, *N*-acetylglucosamine, and *N*-acetylgalactosamine were dissolved in distilled water as standard solutions. The monosaccharide composition of crude HKP was determined using an LC-20AD HPLC system with an Xtimate C18 column (Shimadzu Global Laboratory Consumables Co., Shanghai, China). The column temperature was 30 °C. The injection volume was 20 μL. The flow rate was 1.0 mL/min, and the detection wavelength was set to 250 nm. The mobile phase was a 0.05 M mixed solution that included KDP (A) (pH = 6.70) and ACN (B) (volume ratio = 83:17).

### 2.6. Molecular Weight of HKP

The molecular weight and chain conformational parameters of the HKP sample were determined by HPGPC using size exclusion chromatography combined with multi-angle laser light scattering and refractive index detectors (SEC-MALLS-RI) and calculated according to the Mark–Houwink equation, as previously reported [18]. The weight and number-average molecular weight (Mw and Mn) and polydispersity index (Mw/Mn) for HKP in 0.1 M NaNO_3_ aqueous solution containing 0.02% NaN_3_ at 45 °C were detected on a DAWN HELEOS-II laser photometer (Wyatt Technology Co., Santa Barbara, CA, USA), combined with Shodex OH-pak SB-805 HQ, 804 HQ, and 803 HQ columns (300 × 8 mm, Showa Denko K.K., Tokyo, Japan). Additionally, a differential refractive index detector (Optilab T-rEX, Wyatt Technology Co., Santa Barbara, CA, USA) was connected to obtain the concentration of the fractions and the dn/dc value. The dn/dc value of the sample in 0.1 M NaNO_3_ aqueous solution containing 0.02% NaN_3_ was measured to be 0.141 mL/g. Data were acquired and processed using ASTRA6.1 (Wyatt Technology Co., Santa Barbara, CA, USA).

### 2.7. Fourier Transform Infrared Spectroscopy (FT-IR)

The infrared spectrum of the polysaccharide sample was studied with reference to the KBr tablet pressing method reported by Liu et al. [16] and was modified appropriately. Dried HKP (1 mg) was mixed with KBr crystals (100 mg). This was ground and pressed, then scanned and analysed on an Invenio R infrared spectrometer (Bruker Co., Rheinstetten, Germany) in the range of 400–4000 cm^−1^.

### 2.8. Congo Red Analysis

The conformational transitions of HKP were analysed according to the method described by Tang et al. [18]. First, 1 mL of the HKP sample solution (1 mg/mL) was diluted to an equal ratio with 1 mg/mL Congo red solution. Then, the final concentration of NaOH in the solution was gradually increased from 0.0 M to 0.5 M by gradually adding 1 M NaOH solution. The Congo red solution was mixed with the same concentration of NaOH as used in the control group. The maximum absorption wavelengths of HKP with Congo red solution and NaOH were determined using a UV-1800 double-beam ultraviolet-visible spectrophotometer (Macylab Instruments Inc., Shanghai, China) in the 400–600 nm range. The NaOH concentration was set as the horizontal coordinate (x), and the maximum absorption wavelength was set as the vertical coordinate (y).

### 2.9. Statistical Analysis

All the experimental results were repeated three times and expressed as mean ± standard deviation (SD). Origin 8.1 software (OriginLab, Northampton, MA, USA) was used for graphical processing.

## 3. Results and Discussion

### 3.1. Extraction Yields and Chemical Compositions

The extraction yield of HKP was 3.17 ± 1.30%. This was significantly lower than the polysaccharide content in hemp seeds (10–15%), and the reason may be that the polysaccharide contents in different parts of fruiting bodies were different [8]. The carbohydrate content in HKP was 59.02 ± 5.02%, indicating that carbohydrate was the main component. Additionally, the HKP also contained 3.62 ± 2.13% protein. This may be because the protein content was too high to be removed entirely by multiple enzymatic treatments. Fen et al. [19] reported that the protein content in hemp seeds was very high (72.26%); although the protein content significantly decreased after treatment with protease repeatedly, there was still a tiny amount of protein remaining, which is similar to this study.

### 3.2. Antioxidant Activity of HKP and HKPs

**Figure 1:**
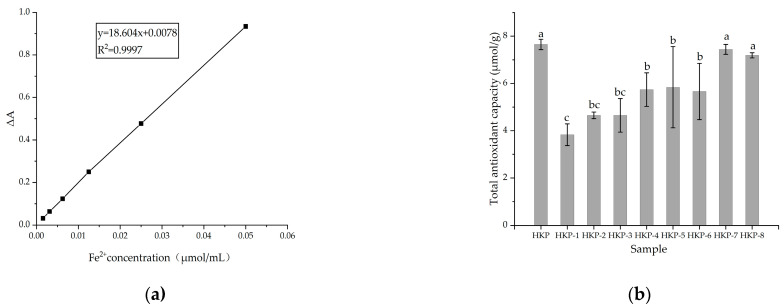
Antioxidant activities of HKP and HKPs. (**a**) Standard curve of T-AOC; (**b**) T-AOC of HKP and HKPs. Note: The values are presented as mean ± SD (n = 3). Different lowercase letters indicate significant differences (*p* < 0.05) between the data within each group.

**Figure 2:**
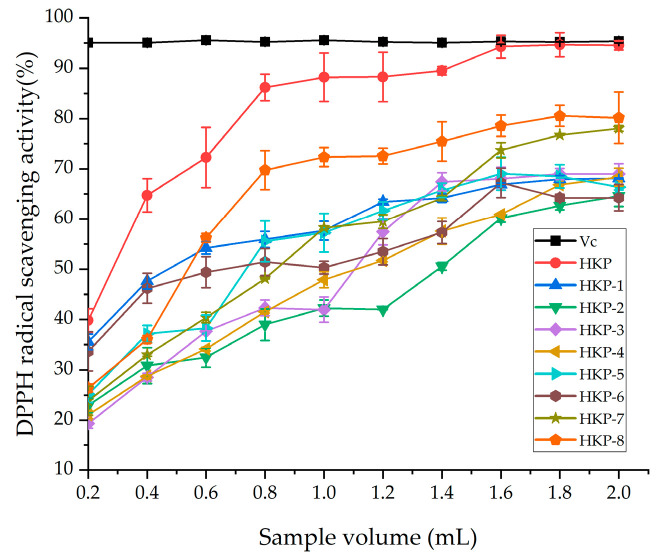
DPPH radical scavenging activity of HKP and HKPs. Note: The data in the Figure 2 are shown as mean ± SD (n = 3).

The antioxidant activities of the HKP and HKPs are shown in Figure 1. Figure 1a shows a standard curve of total antioxidant capacity (T-AOC). The T-AOC of the HKP cells was calculated according to a standard curve. As we can see, HKP displayed the highest Fe^3+^-reducing power (7.65 ± 0.22 μmol/g) at the concentration of 2 mg/mL (Figure 1b). Additionally, HKP-7 and HKP-8 showed high Fe^3+^-reducing power, which was not significantly different from that of HKP (*p* > 0.05). It can be inferred that molecular weight is an essential factor influencing the antioxidant activity of monosaccharides. The DPPH radical scavenging activity of the HKPs was investigated at a concentration of 5.0 mg/mL; the results are summarised in Figure 2. The results showed that as the sample volume increased, the DPPH radical-scavenging activity of the HKPs was constantly enhanced and finally stabilised. Notably, under the same volume experimental conditions (0.2–2.0 mL), the antioxidant activity of HKP was always higher than that of the other HKPs. When the sample volume was above 1.6 mL, the DPPH radical-scavenging rate of HKP was more than 94.30%, almost as high as 5 mg/mL Vc (DPPH radical-scavenging rate of Vc was 95%). HKP showed stronger antioxidant activity than the HKPs, and the molecular weight may not be the only influencing factor. The high antioxidant activity of polysaccharides is attributed to molecular weight, monosaccharide composition, solubility, configuration, etc. [16,20]. The strong antioxidant activities of HKP are the synergistic effects of these factors. Based on the above results, HKP was screened out for subsequent structural identification.

### 3.3. Monosaccharide Composition of HKP

**Figure 3:**
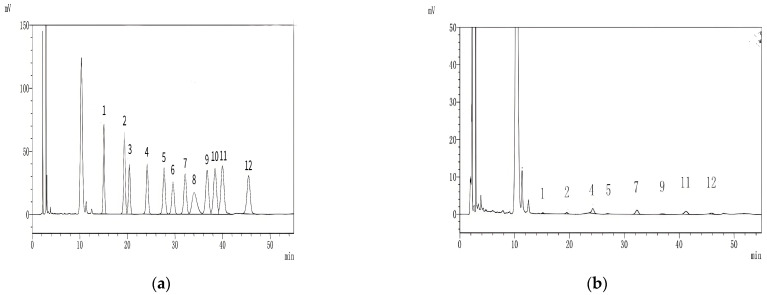
Monosaccharide composition of HKP. (**a**) HPLC chromatograms of monosaccharide standard samples; (**b**) HPLC chromatograms of monosaccharide polysaccharide of HKP. Note: 1—mannose, 2—ribose, 3—rhamnose, 4—glucuronic acid, 5—galacturonic acid, 6—*N*-acetyl-galactose, 7—glucose, 8—*N*-acetyl-galactose, 9—galactose, 10—xylose, 11—arabinose, 12—fucose.

High-performance liquid chromatography (HPLC) was used to determine the monosaccharide composition of the standard and HKP (Figure 3). As shown in Figure 3b, HKP was composed of mannose, ribose, glucuronic acid, galacturonic acid, glucose, galactose, arabinose, and fucose at a molar ratio of 0.96:1.95:8.27:0.98:9.46:1.69:6.10:2.82. Compared with previous studies on hemp seed polysaccharides (HSP), we found that glucose and arabinose were the main components of both polysaccharides. However, HSP are rich in galactose, whereas HKP is rich in glucuronic acid [8,12,14].

Monosaccharide composition is one of the main factors influencing the biological activity of polysaccharides [21,22]. HKP is rich in glucose, which may explain the high antioxidant activity. Glucose is necessary for polysaccharides to exert their antioxidant activities [16]. HPK also contains large amounts of glucuronic acid. Improving the water solubility of HPK enhances its biological activity [23]. It also introduces a large number of electrophilic groups, such as ketones or aldehydes, which enhance the free radical scavenging ability by facilitating the release of hydrogen in the O—H chain [22,24].

### 3.4. Molecular Weight and Chain Conformation of HKP

**Figure 4:**
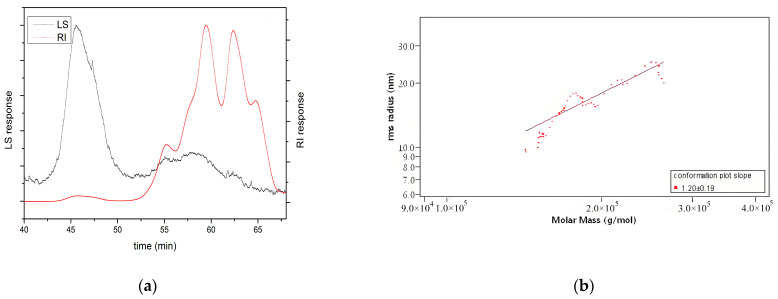
Determination of molecular weight and chain conformation of HKP by HPGPC. (**a**) Molecular weight; (**b**) chain conformation.

Undoubtedly, the molecular weight and conformation of polysaccharides also affect their biological activity, although the specific mechanism is not fully understood [25,26,27]. Among them, the molecular weight of polysaccharides is considered the most important factor affecting antioxidant activity [28]. Low-molecular-weight polysaccharides are better at accepting and neutralising free radicals due to their higher number of reductive hydroxyl group terminals (per unit mass basis) [22]. However, high-molecular-weight polysaccharides have a more compact structure, leading to stronger intramolecular hydrogen bonds, which limit the number of hydrogen groups [29]. In this study, as shown in Table 1, the molecular weight (MW) of HKP was 4.21 ± 0.12 kDa, detected by SEC-MALLS-RI. Also, the polydispersity (Mw/Mn) was 4.39 ± 0.01, indicating a wide molar mass distribution of HKP. In addition, as shown in Figure 4a, large and wide small peaks were detected using laser light scattering (LLS). Two large and three small peaks were detected using the refractive index (RI), with two large and one small peak in the high-retention-time region and two small peaks in the low-retention-time region. The results of the two detection methods suggested the presence of high-molecular-weight components and a few aggregates in the HKP. These results revealed that HKP is a polysaccharide with a wide mass distribution and low molecular weight. Low molecular weight is one of the reasons why HKP has higher antioxidant activity than other HKPs. This result is consistent with the findings of other researchers [29,30].

In terms of polysaccharide conformation, the Mark–Houwink equation is commonly used to infer the chain conformation of polysaccharides. According to the Mark–Houwink equation, when the slope value is lower than 0.33, between 0.5 and 0.6, and from 0.6 to 1.0, the polysaccharide in solution is deemed to be in a tight uniform spherical conformation, a random coil conformation, and a rigid rod conformation, respectively [27]. Figure 4b shows log-log plots of <S^2^>^1/2^ versus Mw of HKP. The slope value was calculated to be 1.20 ± 0.19, which meant that the conformation of HKP could not be determined.

### 3.5. FT-IR Spectrum of HKP

**Figure 5:**
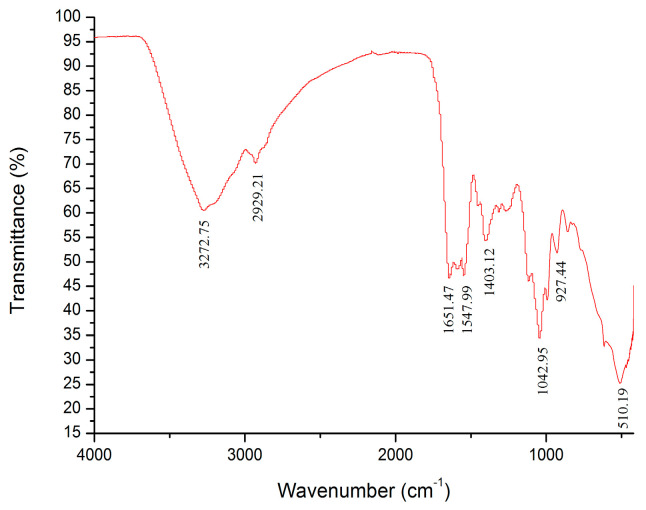
Infrared spectrum of HKP.

The characteristic absorption spectra of HKP determined by IF-IR spectrometry are shown in Figure 5. The absorption peaks of HKP were obvious in the range of 4000–400 cm^−1^. The strong absorption peak at 3272.75 cm^–1^ was owing to the stretching vibration of the hydroxyl group (O–H bond) [13,27]. A weak absorption peak caused by C–H stretching vibration was observed at 2929.21 cm^−1^ [13,16,27,31]. There was no absorption peak between 2500 and 1900 cm^−1^, indicating no accumulation of double or triple bonds [15]. An absorption peak at 1651.47 cm^−1^ was probably ascribed to asymmetric stretching vibration of the carboxyl (–COOH) C=O [32,33,34]. This suggests that uronic acid is present in HKP cells. This conclusion is consistent with the above results for the HKP monosaccharide composition. There was an absorption peak (1547.99 cm^−1^) between 1657 and 1500 cm^−1^, indicating the stretching vibration of N–H [35]. The absorption peak (1403.12 cm^−1^) between 1407 and 1200 cm^−1^ was attributed to the stretching vibration of C–H [36,37]. The IR spectrum from 800 to 1300 cm^−1^ is considered to be the “fingerprint” region of carbohydrates, which is correlated with the conformation and surface structure of polysaccharide molecules. An absorption peak appeared at 1042.95 cm^−1^, revealing that HKP had stretching vibration of the C–O–C bond and contained a pyranose ring [35,38,39]. The absorption peaks at 927.47 cm^−1^ and 510.19 cm^−1^ were owing to asymmetric stretching vibration and symmetrical stretching vibration of the pyranose ring, respectively [16,21,40]. From the above analysis of the infrared spectrum, it was deduced that HKP was an acidic polysaccharide with a β-configuration.

### 3.6. Analysis of Congo Red

**Figure 6:**
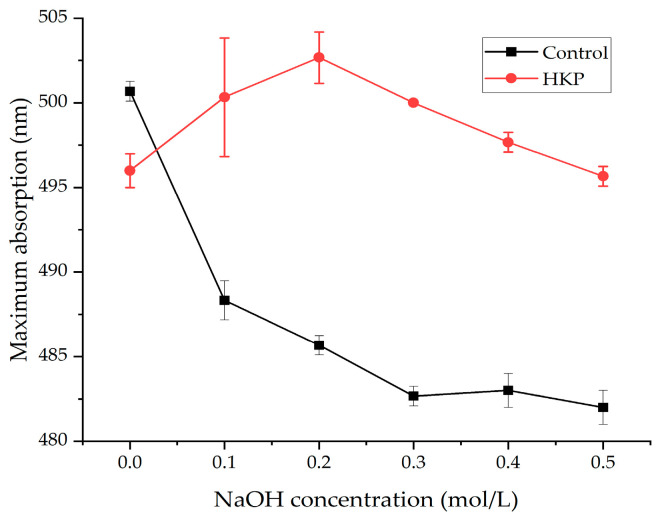
Congo red analysis. Note: The data are shown as mean ± SD (n = 3).

Polysaccharides with a triple helical structure can form a complex with Congo red at varying alkaline solution concentrations, generating a bathochromic shift in the absorption maximum compared to pure Congo red in NaOH solution [15]. As the concentration of NaOH increased, the destruction of the triple helical structure caused a decrease in the maximum absorption. The maximum absorption wavelength of the HKP–Congo red complexes was red-shifted compared with that of the Congo red solution (Figure 6). When the concentration of NaOH increased from 0.0 mol/L to 0.2 mol/L, the maximum absorption shifted to a long wave, indicating that HKP samples could form a complex with Congo red and the HKP sample had regular helical conformation. As the NaOH concentration increased, the maximum absorption wavelength decreased, suggesting that the spiral structure of the polysaccharide disintegrated and changed into an irregular coil form. HKP has a triple helical structure.

Interestingly, some researchers have pointed out that polysaccharides with a molecular weight greater than 90 kDa can form triple helical structures [41]. Many previous studies support this viewpoint [42,43,44]. However, HKP has a triple helical structure despite its molecular weight of only 4.21 ± 0.12 kDa. This result is similar to that reported by Guo et al. [45] and is not commonly observed. This may be because the special structure of HKP enhances the interaction forces between its glycosidic bonds, intramolecular hydrogen bonds, and intermolecular hydrogen bonds to form a triple helix structure.

## 4. Conclusions

HKPs were isolated from shelled hemp seeds. The Fe^3+^-reducing power of the HKP was 7.65 ± 0.22 μmol/g, and it also possessed the highest DPPH radical-scavenging rates (94.30 ± 2.27%) compared to other HKPs. It was mainly composed of mannose, ribose, glucuronic acid, galacturonic acid, glucose, galactose, and fucose in a molar ratio of 0.96:1.95:8.27:0.98:9.46:1.69:6.10:2.82. Among them, glucose content was the highest (29.35%), followed by glucuronic acid (25.66%) and arabinose (18.93%). Also, HKP was an acidic polysaccharide with wide molar mass distribution and low molecular weight (4.21 ± 0.12 kDa). Based on the characteristic detection results of HKP, it was inferred that HPK had a β-configuration and a triple helix conformation. The high antioxidant activity of HKP may be related to the molecular weight, monosaccharide composition, solubility, and configuration. In the future, it will be necessary to purify HKP and further study its secondary and tertiary structures and its different biological activities, such as hypoglycaemic, anti-tumour, and immunostimulatory activities.

## Figures and Tables

**Table 1:** Molecular weight of HKP.

Mw (kDa)	Mn (kDa)	Polydispersity (Mw/Mn)
4.21 ± 0.12	0.96 ± 0.09	4.39 ± 0.01

Note: Values represent the mean ± SD (n = 3).

## Data Availability

The original contributions presented in the study are included in the article, further inquiries can be directed to the corresponding author.
